# A short communication of pain outcomes following a pharmacist-delivered alcohol and opioid use reduction intervention

**DOI:** 10.1016/j.dadr.2026.100418

**Published:** 2026-02-27

**Authors:** Gerald Cochran, Grace Broussard, Yingjia Wei, Craig Field, Adam J. Gordon, Kenneth C. Hohmeier

**Affiliations:** a University of Utah, Division of Epidemiology, 295 Chipeta Way, Salt Lake City, UT 84108, United States; b University of Texas at El Paso (UTEP), Department of Psychology, 500 W. University Avenue, El Paso, TX 79968, United States; c University of Texas, El Paso, University of Tennessee Health Science Center, 301 S. Perimeter Park Drive, Suite 220, Nashville, TN 37211, United States

**Keywords:** Brief medication management intervention, Alcohol, Opioid, Community pharmacy, Opioid and alcohol co-use

## Abstract

**Background:**

Co-use of alcohol and opioid medications increases risk for sedation, respiratory depression, and overdose, yet remains common among patients prescribed opioids. The Alcohol Brief Intervention–Medication Therapy Management (ABI-MTM) intervention was developed for delivery by community pharmacists and demonstrated feasibility, acceptability, and preliminary reductions in co-use. Given possible pain-related motives for co-use, this exploratory secondary analysis assessed whether ABI-MTM affected pain symptomatology.

**Methods:**

This study utilized data from a randomized trial of 44 community pharmacy patients from 25 pharmacies prescribed opioids and who reported alcohol co-use. Participants were randomized to ABI-MTM or standard medication counseling (SMC). Pain intensity/interference were assessed at baseline, 2-, and 3-months using the Brief Pain Inventory–Short Form. Analyses included descriptive statistics, Cohen’s d effect sizes, and mixed-effects models comparing pain across conditions and timepoints.

**Results:**

Pain scores did not differ between groups (p > 0.05). For pain intensity, ABI-MTM and SMC showed similar baseline means (4.31 vs. 5.05), decreased modestly at 2-months (2.80 vs. 3.74), and returned to baseline levels at 3-months (4.21 vs. 4.83). Pain interference followed a comparable pattern, with ABI-MTM and SMC starting similarly (4.83 vs. 5.07), decreasing modestly at 2-months (3.19 vs. 3.87), and returning near baseline at 3-months (4.92 vs. 4.42). Effect sizes between group differences were small (Cohen’s d≤0.33). Mixed-model analyses showed no significant treatment effects on pain intensity/interference across time (p > 0.05).

**Conclusions:**

This underpowered study found no evidence of pain differences between ABI-MTM and SMC, tentatively suggesting possible alcohol-opioid co-use improvements associated with the intervention without worsening pain.

## Highlights

• The pharmacist intervention was associted with reduced alcohol and opioid co-use without increasing pain.
• Pain scores remained stable across both groups.
• Community pharmacists can play a meaningful role in addressing opioid and alcohol co-use.

## 1 Introduction

Co-use of alcohol and opioid medications increases the risk of sedation, respiratory depression, and fatal overdose for patients due to their combined respiratory depressant effects (CDC, 2025, FDA., 2018, Kuerbis et al., 2014). Despite significant risks, alcohol and opioid co-use are prevalent in clinical and community settings (Esser et al., 2019, Hartzler et al., 2010, Hser et al., 2017). One example comes from our work in screening patients dispensed opioid medications in community pharmacies in multiple states over the past decade. Our data has consistently shown 20–30% of community pharmacy patients dispensed opioid pain medications report concurrent high-risk drinking (Cochran et al., 2016, Cochran et al., 2021, Cochran et al., 2022, Cochran et al., 2019, Cochran et al., 2017).

Co-use of alcohol and opioid medications may be based in efforts by patients to manage pain symptomology (Witkiewitz and Vowles, 2018). This possible link is rooted in the analgesic properties of both alcohol and opioids. While some evidence may be mixed regarding quantity of alcohol needed to achieve specific levels of analgesia, meta-analytic evidence supports alcohol’s ability to control pain (Thompson et al., 2017), and the primary indication for opioid medications is treatment of painful conditions (Dowell et al., 2022). Despite these facts, co-use in all forms is highly discouraged (CDC, 2018, NIAAA, 2007). To increase intervention capacity of pharmacists dispensing opioids to patients who also report alcohol use, our team recently completed a developmental study/randomized controlled trial (NCT05599672) testing pharmacist-delivered Alcohol Brief Intervention-Medication Therapy Management (ABI-MTM) compared to standard medication counseling (SMC) (Cernasev et al., 2023, Hohmeier et al., 2025). As the last point of care prior to opioid dispensation, pharmacists may have a unique opportunity to assist patients prevent or reduce co-use risks.

ABI-MTM is a brief medication management intervention that identifies patients dispensed an opioid medication who also report concurrent alcohol use. In connection with our developmental trial’s results that showed acceptability and feasibility of the intervention, our findings also demonstrated provisional efficacy that a greater portion of those who received ABI-MTM reduced ≥ 30% drinks per drinking day and/or daily morphine milligram equivalents (59.1%) compared to SMC (45.5%) at 3-months post-baseline (Hohmeier et al., 2025). These observed effects of the ABI-MTM intervention are consistent with effects of previous research for alcohol (Bien et al., 1993, Kaner et al., 2018, Moyer et al., 2002, Vasilaki et al., 2006) and opioid medication-focused (Bohnert et al., 2016, Cochran et al., 2019, McCauley et al., 2013, Zahradnik et al., 2009) brief interventions.

Reductions in opioid and/or alcohol co-use, nevertheless, have the potential for unintended consequences. Given that alcohol consumption among patients dispensed opioid pain medications may be intended by those patients to enhance pain control, reduction of either may produce increased pain. Unmanaged pain following alcohol and/or opioid medication reductions may lead to return to use and possible overuse as individuals attempt to regain pain control. Severe unmanaged pain can also engender suicidal ideation and risk (Ratcliffe et al., 2008, Torino et al., 2025). Sudden return to opioid use after decreases or periods of abstinence can increase overdose risk. It is therefore critical to elucidate the impact of ABI-MTM on pain to understand potential risk. For this short communication, we examined differences in pain intensity and interference between ABI-MTM and SMC and explored differences in pain across time among these groups.

## 2 Methods

### 2.1 Study design and participants

This exploratory secondary analysis used data from our recently completed developmental study. Details of the parent trial methods are described elsewhere (Cernasev et al., 2023) and are briefly described herein. The parent trial included adults recruited from 25 community pharmacies in Utah and Tennessee. Prescreening asked participants to affirm they were ≥ 18 years old, not receiving cancer treatment, and not pregnant. Patients were also asked about their opioid regimen and their current alcohol use (using the Alcohol Use Disorders Identification Test-Consumption [AUDIT]-C) (Bradley et al., 2007) to confirm opioid use overlapped with alcohol use. Since co-use is never recommended, we recruited with equal condition stratification individuals reporting low- and high-risk drinking (an AUDIT-C score of ≥4 or in men and ≥3 or more in women) (Bradley et al., 2007).

Screening verified prescreening criteria and excluded participants who lacked two collateral contacts, did not have a reliable phone, filled only buprenorphine prescriptions, planned extended travel in the next three months, or reported a psychotic/manic episode within 30 days. Participants meeting eligibility criteria were asked to provide informed consent, were enrolled into the study, completed a baseline assessment, and were randomized utilizing a 1-to-1 randomization allocation ratio (stratified by low vs high risk drinking status) to ABI-MTM (n = 22) or SMC (n = 22).

### 2.2 Intervention conditions

The control arm included SMC plus enhanced information, delivered by a licensed doctoral-level pharmacist. All SMC participants were offered a pharmacist counseling session meeting federal and state requirements and received follow up safety information on alcohol and opioid co-use. This condition was designed to be equally yoked with ABI-MTM to control for attention.

ABI-MTM is a pharmacy-based medication management approach integrated with a brief motivational intervention (Beich et al., 2003, Bertholet et al., 2005, Cernasev et al., 2023). It targets (1) reducing alcohol use during the prescribed opioid therapy, (2) decreasing opioid medication use if the individual was not interested in reducing alcohol use, or (3) both. The intervention consisted of a single phone session with a 7-day telephone follow-up booster session. Importantly, given the brief nature of the single session and booster session, this intervention was specifically designed to address the named target behaviors, i.e., co-use. It was not intended to address pain/perceptions, mental health, or well-being of the patient—although—improvements in such areas may possibly follow improvements in alcohol and opioid medication co-use.

### 2.3 Study Assessments and Variables

Based on data from the baseline, 2-, and 3-month study assessments, the parent study demonstrated acceptability, feasibility, and preliminary efficacy of ABI-MTM. Preliminary efficacy was measured by achieving ≥ 30% reductions in drinks per drinking day (DDD) and/or morphine milligram equivalents (MME). For this secondary exploratory analysis, the primary outcome was pain level, measured at the baseline, 2-, and 3-month assessments using the brief pain inventory short form (Atkinson et al., 2010, Gjeilo et al., 2007, Keller et al., 2014, Mendoza et al., 2006). This measure assessed participants’ pain intensity and interference at each timepoint, with scores ranging from one to ten. In addition, given the close association between pain and depression, we also conducted an exploratory analysis of the impact of the intervention condition on depression (Levis et al., 2019, Patient Health Questionnaire-9) to identify any signals of improvement.

We also report baseline assessments, including rates of opioid use disorder (Denis et al., 2015), health functioning (Atkinson et al., 2010, Gjeilo et al., 2007, Mathias et al., 2011, Mendoza et al., 2006, Ware, 2000), depression (cutoff of 5 indicating mild or moderate depression) and post-traumatic stress disorder ([PTSD] Bovin et al., 2021, Primary Care Posttraumatic Stress Disorder screener, a cutoff of 3 indicating a positive screen), and sociodemographic characteristics (PhenxToolKit, 2015).

### 2.4 Analyses

To describe the population characteristics and compare pain at each timepoint, we utilized descriptive statistics, T-tests, and Chi-Square tests. We utilized the same tests exploring differences in depression at each timepoint. We calculated Cohen’s d to assess the magnitude of differences in pain at the assessment timepoints. We performed unadjusted and adjusted mixed effect regression analysis to assess differences at the 2- and 3-month assessments compared to baseline for level of pain intensity and interference within and between groups across time. In the adjusted analyses, we included age, sex, educational status, employment status, morphine milligram equivalents across time, and drinks per drinking day across time as covariates. We utilized the same tests exploring changes in depression across time.

## 3 Results

The assessment of baseline opioid use disorder, health functioning, mental health, and sociodemographic characteristics showed no statistically significant differences between ABI-MTM and SMC recipients (results not shown, see Hohmeier et al., 2025). Overall, most participants were from Utah (77.3%, n = 34), averaged 56 years old (standard deviation [SD]=15.3), and were female 65.9% (n = 29). Participants were White (95.5%, n = 42), had completed high school or more (95.5%, n = 42), and had health insurance (95.5%, n = 42). Half of participants reported working part or full time (50%, n = 17).

More than one out of ten participants screened positive for opioid use disorder (13.6%, n = 6); 29.6% (n = 13) screened positive for moderate/severe depression, and 38.6% screened positive for PTSD. In addition, 20.5% (n = 9) of participants indicated a having a physical role limitation, and 36.4% reported having poorer general health (Stewart, Ware, 1992) (n = 16) than the general population.

### 3.1 Pain outcomes

Table 1 displays patient reported pain levels at baseline, 2-, and 3-month timepoints. Pain intensity and interference scores did not significantly differ between ABI-MTM and SMC participants at any time point (*p* > 0.05: *intensity*: baseline ABI-MTM M=4.31, SD=2.15 vs. SMC M=5.05, SD=2.36, 2-months ABI-MTM M=2.80, SD=2.93 vs. SMC=3.74, SD=2.67, and 3-months ABI-MTM M=4.21, SD=2.37 vs. SMC M=4.83, SD=2.37; *interference*: baseline ABI-MTM=4.83, SD=2.35 vs. SMC M=5.07, SD=2.79, 2-months ABI-MTM M=3.19, SD=3.49 vs. SMC M=3.87, SD=2.82, and 3-months ABI-MTM M=4.92, SD=2.86 vs. SMC M=4.42, SD=2.68). Small effect sizes were observed for all differences (Cohen’s d≤0.33). Similarly, we did not observe any significant differences for depression scores at any timepoint (p > 0.05, results not shown).

**Table 1:** Descriptive Analyses of Pain Intensity and Interference by Treatment Condition at Baseline, 2-, and 3-months.

**Variable**	**ABI-MTM**	**SMC**			
**Intensity**	**Mean (SD)**	**Mean (SD)**	**t (df)**	**p**	**Cohen's d**
Baseline	4.31 (2.15)	5.05 (2.36)	-1.07 (41)	0.291	0.33
2 months	2.80 (2.93)	3.74 (2.67)	-1.06 (38)	0.297	0.33
3 months	4.21 (2.37)	4.83 (2.37)	-0.82 (39)	0.415	0.26
**Interference**					
Baseline	4.83 (2.35)	5.07 (2.79)	-0.31 (40)	0.761	0.09
2 months	3.19 (3.49)	3.87 (2.82)	-0.68 (36)	0.503	0.21
3 months	4.92 (2.86)	4.42 (2.68)	0.58 (39)	0.565	-0.18

Table 2 shows mixed model results for pain levels across time between groups. Although descriptive analyses showed pain scores slightly lowered at 2-months, unadjusted mixed model results showed no statistically significant differences at the 2- or 3-months follow up assessment for the association of treatment condition with intensity (2-months B = −0.19, 95% CI [-1.94, 1.57], p = 0.83, 3-months B = 0.09, 95% CI [-1.44, 1.63], p = 0.90) or interference (2-months B = −0.63, 95% CI [-2.56, 1.30], p = 0.52, 3-months B = 0.52, 95% CI [-0.80, 1.83], p = 0.44). Similarly, adjusted models showed no statistically significant differences at the 2- or 3-months follow-up assessment in intensity (2-months B = −0.34, 95% CI [-2.13, 1.45], p = 0.71, 3-months B = −0.01, 95% CI [-1.58, 1.55], p = 0.99) or interference (2-months B = −0.77, 95% CI [-2.71, 1.17], p = 0.43, 3-months B = 0.49, 95% CI [-0.88, 1.88], p = 0.48). Finally, using these same mixed models, we did not observe any significant differences over time for changes in level of depression (p > 0.05, results not shown).

**Table 2:** Unadjusted and Adjusted Mixed Model Analyses of the Alcohol Targeted Brief Intervention Medication Therapy Management Intervention (ABI-MTM) on Pain Intensity and Interference.

Effect	*Unadjusted Estimate*	*95% CI*	*P-value*
***Intensity***			
ABI-MTM - 2 months	-0.19	[-1.94, 1.57]	0.83
ABI-MTM - 3 months	0.09	[-1.44, 1.63]	0.90
***Interference***			
ABI-MTM - 2 months	-0.63	[-2.56, 1.30]	0.52
ABI-MTM - 3 months	0.52	[-0.80, 1.83]	0.44
	Adjusted Estimate*	*95% CI*	*P-value*
***Intensity***			
ABI-MTM - 2 months	-0.34	[-2.13, 1.45]	0.71
ABI-MTM - 3 months	-0.01	[-1.58, 1.55]	0.99
***Interference***			
ABI-MTM - 2 months	-0.77	[-2.71, 1.17]	0.43
ABI-MTM - 3 months	0.49	[-0.88, 1.87]	0.48

⁎ Models were adjusted for morphine milligram equivalents, drinks per drinking day, sex, age, educational status, and employment status

## 4 Discussion

Our parent developmental study showed promising results indicating that ABI-MTM may reduce co-use (Hohmeier et al., 2025). However, it is critical to examine if the pain intensity and interference were simultaneously impacted given reductions in co-use—increasing risk for unmanaged pain, return to use, and overuse. This underpowered secondary analysis showed no statistically significant differences within and between ABI-MTM and SMC groups, with small effects for differences between groups, for pain interference and intensity, suggesting ABI-MTM may be associated with co-use improvements without potentially being related to differential impact on pain among intervention recipients.

Community pharmacists are increasingly involved in chronic care (Rahayu et al., 2021) given their doctoral level training (Mott et al., 2024), ease of access for patients across geographic regions (Berenbrok et al., 2022), and expanding scope via collaborative care agreements or standard of practice (Adams and Weaver, 2021, Baylis et al., 2025). With continued predictions of physician shortages over the coming decades, pharmacist’s involvement in addressing known care gaps is one potential solution with the promise of scale across the U.S. (Walensky and McCann, 2025). These results possibly support including community pharmacists on care teams to improve safe, effective medication use, without introducing unintended harms to patients (i.e., pharmacist-facilitated reductions in co-use worsening pain scores) (Percy et al., 2023).

Future research could explore why ABI-MTM may not increase pain among those reducing alcohol and opioid co-use, which may relate to hyperalgesia. Opioid medication overuse is linked to possible hyperalgesia and resultant heightened sensitivity to pain (Cahill and Taylor, 2017, Chapman et al., 2010, De Aquino et al., 2024, Manchikanti, 2007). Similar findings for hyperalgesia and heightened pain sensitivity have been documented for overuse of alcohol in both preclinical and clinical research studies (Cucinello-Ragland and Edwards, 2021, Fu et al., 2015, Vigorito and Chang, 2024). Our findings of no differential impact on pain possibly may possibly align with research suggesting that reducing opioid overuse may improve pain outcomes for patients (Cochran et al., 2019). Future research could clarify the interrelationship between pain, opioids, and alcohol.

## 5 Limitations

While this secondary data analysis has several strengths—it nevertheless includes limitations that should be considered. First, this study was not specifically designed to answer questions about the impact of the intervention on pain outcomes. However, given our longitudinal randomized design along with validated measures, this study has strong internal validity. Future research designs ideally will more intentionally account for the impact pain, opioid use, alcohol use, and mental/physical health related to the study intervention. Also, the parent study has a limited sample in terms of size and demographic characteristics, limiting external validity. For instance, based on the numbers of participants in this study, this sample possessed 20–30% power for achieving a 1-point difference between groups in pain. Thus, while this study and its parent study provide possible insight into tentative signals, it is critical that subsequent studies in this area should seek to enroll a larger number of patients in a powered trial that has increased variability of sociodemographic characteristics to enhance generalizability.

## 6 Conclusion

Over three months, receipt of ABI-MTM was not associated with increased or differential levels of pain. These findings suggest a durable effect of ABI-MTM without differential pain-related consequences.

## CRediT authorship contribution statement

**Hohmeier Kenneth:** Writing – review & editing, Investigation, Funding acquisition, Conceptualization. **Gordon Adam:** Writing – review & editing, Funding acquisition, Conceptualization. **Gerald Cochran:** Writing – review & editing, Writing – original draft, Supervision, Project administration, Investigation, Funding acquisition, Formal analysis, Conceptualization. **Grace Broussard:** Writing – review & editing, Writing – original draft, Project administration, Formal analysis, Data curation. **Craig Field:** Writing – review & editing, Investigation, Conceptualization. **Yingjia Wei:** Writing – review & editing, Writing – original draft, Formal analysis, Data curation.

## Funding

This work was supported by the 10.13039/100000002National Institutes of Health (NIAAA R34AA029447).

## Declaration of Competing Interest

The authors declare the following financial interests/personal relationships which may be considered as potential competing interests: Gerald Cochran reports financial support was provided by The University of Utah School of Medicine. Gerald Cochran reports a relationship with National Institute on Alcohol Abuse and Alcoholism that includes: funding grants. If there are other authors, they declare that they have no known competing financial interests or personal relationships that could have appeared to influence the work reported in this paper.
